# COVID-associated complications after reconstructive breast surgery: a retrospective cohort study

**DOI:** 10.1007/s10549-023-07064-1

**Published:** 2023-07-29

**Authors:** J. M. Bubberman, J. Claessen, M. M. W. Feijen, M. A. J. Meesters-Caberg, S. M. J. Van Kuijk, R. R. W. J. Van der Hulst, S. M. H. Tuinder

**Affiliations:** 1 Department of Plastic, Reconstructive and Hand Surgery, Maastricht University Medical Center, P.O. Box 5800, 6202 AZ Maastricht, The Netherlands; 2 GROW – School for Oncology and Developmental Biology, Maastricht University Medical Center, Maastricht, The Netherlands; 3 Faculty of Health, Medicine, and Life Sciences, Maastricht University, Maastricht, The Netherlands; 4 Department of Plastic, Reconstructive and Hand Surgery, Zuyderland Medical Center, Sittard-Geleen, The Netherlands; 5 Department of Clinical Epidemiology and Medical Technology Assessment, Maastricht University Medical Center, Maastricht, The Netherlands

**Keywords:** Breast cancer, Breast reconstruction, COVID-19, Postoperative complications

## Abstract

**Purpose:**

The COVID pandemic significantly influenced reconstructive breast surgery regimens. Many surgeries were cancelled or postponed. COVID entails not only respiratory, but also coagulative symptoms. It, therefore, potentially increases the risk of postoperative complications. The incidence of perioperative COVID infection and its influence on postoperative recovery after reconstructive breast surgery is still unknown.

**Methods:**

This dual center retrospective cohort study included patients that underwent reconstructive breast surgery between March 2020 and July 2021. Post-mastectomy autologous or implant-based breast reconstruction (ABR; IBR), as well as post-lumpectomy oncoplastic partial breast reconstruction (PBR) were eligible. Patient data were extracted from electronic medical records. Data regarding COVID-19 infection was collected through a questionnaire. The primary outcome was complication rate.

**Results:**

The ABR, IBR and PBR groups consisted of 113 (12 COVID-positive), 41 (2 COVID-positive) and 113 (10 COVID-positive) patients. In the ABR and PBR groups, postoperative complications occurred significantly more often in patients with perioperative COVID-infection. Especially impaired wound healing occurred significantly more often in the ABR and PBR breasts, but also at the donor site of ABR patients with perioperative COVID.

**Conclusion:**

Perioperative COVID-infection increases susceptibility to complicated wound healing after reconstructive breast surgery. A possible explanation lies in the dysregulation of haemostasis by the virus, and its direct effects on microvasculature. A hypercoagulable state results. We recommend to postpone elective breast surgery for 4–6 weeks after COVID-19 infection. Also, precautionary measures remain important to minimize the risk of perioperative COVID-19 infection.

**Supplementary Information:**

The online version contains supplementary material available at 10.1007/s10549-023-07064-1.

## Introduction

Breast reconstruction is an integral part of breast cancer care. Restoring the aesthetics of the female breast after a mastectomy or lumpectomy leads to greater patient satisfaction and improves quality of life [1]. The coronavirus disease 2019 (COVID-19) pandemic greatly influenced surgical regimens. Reconstructive breast surgery was often postponed or cancelled as a transmissibility precaution and because of reduced capacity [2–4].

Several studies aimed to elucidate the consequences of perioperative COVID-infection. The CovidSurg Collaborative reported increased morbidity and mortality after surgery in COVID-positive patients [5]. Furthermore, cancer patients seem prone to suffer from a more complicated disease course if they contract COVID, and undergoing oncological surgery seems to induce more severe disease manifestations [6–8].

In patients with perioperative COVID-infection, the most frequently observed symptoms are respiratory or coagulative [9]. This could increase risks of postoperative thrombo-embolic events, would dehiscence, and impaired flap perfusion after breast reconstruction [10]. However, the topic has not been studied extensively. Therefore, valid evidence is currently lacking.

While the peaks of the COVID-19 pandemic lie in the past, the virus is still present and could pose risks for surgical patients [11]. Hence, understanding how COVID influences postoperative recovery remains important. Current knowledge about the incidence of perioperative COVID-infection, and its influence on postoperative recovery after reconstructive breast surgery is still limited.

In this cohort study, we aim to estimate the risks associated with COVID-infection in patients undergoing reconstructive breast surgery. With this knowledge, timing of surgery and postoperative care can be optimized.

## Methods

A retrospective cohort study was conducted. Patients that underwent breast reconstructive surgery between March 2020 and July 2021 in Maastricht University Medical Center or Zuyderland medical center were included. The start of the cohort coincides with the beginning of the COVID-19 pandemic in the Netherlands, and the end of the cohort is when the majority of the Dutch population was vaccinated. The local ethics committee and institutional review boards gave approval (METC 2021-2765).

Eligible post-mastectomy reconstruction types were abdominal as well as non-abdominal based autologous breast reconstruction (ABR), and implant-based breast reconstruction (IBR). Eligible post-lumpectomy or partial breast reconstruction (PBR) types were oncoplastic reduction techniques as well as locoregional (advancement) flaps. Patients needed to have either had an eligible post-mastectomy or post-lumpectomy reconstruction type. Patients who were aged below 18, illiterate or cognitively impaired were excluded from participation. All patients gave informed consent.

All patients underwent obligatory COVID polymerase chain reaction (PCR) testing < 48 h prior to their surgery according to local protocols. When positive, the surgery was delayed by two weeks, after which the PCR was repeated when symptoms persisted.

Patient data was extracted from electronic medical records and comprised general health-related characteristics (age, BMI, smoking, comorbidities), breast cancer history and treatment (radiotherapy, chemotherapy, immunotherapy, endocrine therapy, prior breast surgery), breast reconstruction details (type, laterality, timing [defined as primary in immediate reconstructions; and secondary or tertiary in delayed reconstructions without and with previous alternative reconstruction, respectively], duration, ischemia time, hospital stay), and complications (intra- and postoperative). Data regarding COVID-19 infection was collected through a structured interview either via telephone or with an online survey. Non-responders were reminded up to two times. Patients were defined ‘COVID-positive’ if they had had a confirmed COVID-infection between six weeks prior to and six weeks after surgery by PCR testing. These cut-offs were chosen based on the estimated recovery time after COVID-infection and the estimated time for surgical wound healing (both up until six weeks). The researchers were blinded to the patients COVID-status during data collection and thereby unbiased.

The primary outcome was complication rate. Complications were divided into intraoperative and postoperative complications for all surgical sites. Major complications were defined as requiring surgical management under local or general anesthesia, infection requiring admission for IV antibiotics, or surgical site complications resulting in a chronic wound requiring prolonged (> 3 months) specialized wound care (such as hyperbaric oxygen therapy or consultation of a wound care specialist). The following individual complications were analyzed: impaired wound healing (> 6 weeks), infection, seroma, hematoma, fat necrosis), and mastectomy skin flap necrosis. All complications were clinically diagnosed by the senior responsible plastic surgeon and defined as minor when managed conservatively.

Data were analyzed using IBM SPSS Statistics version 28 (Armonk, NY: IBM Corp.). Normally and non-normally distributed data were presented as mean ± standard deviation or as median [25th and 75th percentile]; and compared using the independent-samples t-test or the Mann–Whitney *U* test, respectively. Nominal data were presented as absolute counts and proportions, and compared using Pearson’s chi square or Fisher’s exact test as appropriate. The primary and secondary outcome measures were compared using logistic regression analysis. Crude and adjusted odds ratios (OR) with 95% confidence intervals (CI) and p-values were calculated. Data were adjusted for clinically meaningful baseline differences between COVID-19 status groups, determined separately for the ABR, IBR and PBR strata. Due to the relatively low incidence of complications overall, multilevel modelling to adjust for paired data (i.e. two breasts in one patient) was deemed too demanding of that data. P < 0.05 was considered statistically significant.

## Results

### Baseline

In the ABR group, 12 patients were COVID-positive perioperatively (7 preoperative, 5 postoperative). Their mean age was 51.3 years; mean BMI was 27.1. The majority of the mastectomies were for oncological reasons (67.9%); and 34.8% (COVID-negative) versus 27.8% (COVID-positive) of the breasts were irradiated pre-reconstruction. The COVID-negative patients more often had had chemotherapy compared to COVID-positive (65.3% vs. 41.7%). Other characteristics were similar.

In the IBR group, two patients were COVID-positive, both postoperative. Their mean age was 39.5; mean age of the total group was 51.0 years. The two COVID-positive patients had a slightly higher mean BMI than the COVID-negative patients (26.0 vs. 24.3). Comparable to the ABR group, 72.7% of mastectomies were for oncological reasons. Radiotherapy was indicated in 14.5% IBR breast postoperatively.

In the PBR group, 10 patients were COVID-positive perioperatively (4 preoperative, 6 postoperative). Their mean age was 61.9 years. The mean BMI in the COVID-positive patients was higher than in the COVID-negative patients (29.3 vs. 26.6). Of all breasts, 74.6% were irradiated (postoperatively). Other characteristics were comparable. All patient characteristics are presented in Table 1.

**Table 1:** Baseline characteristics

	ABR (*N* = 113)		IBR (*N* = 41)		PBR (*N* = 113)	
	COVID- negative	COVID- positive	COVID- negative	COVID- positive	COVID- negative	COVID- positive
Patients/breasts	101/141	12/18	39/52	2/4	103/121	10/13
Mean age ± SD, yr	51.4 ± 9.7	50.0 ± 9.5	51.6 ± 13.3	39.5 ± 16.3	61.8 ± 11.1	62.9 ± 12.5
BMI ± SD, kg/m^2^	27.1 ± 4.4	26.9 ± 2.6	24.3 ± 3.8	26.0 ± 3.1	26.6 ± 4.2	29.3 ± 4.0
Smoking	2 (2.0)	0	5 (12.8)	0	11 (10.7)	0
Diabetes	1 (1.0)	0	0	0	6 (5.8)	0
Hypertension	14 (13.9)	1 (8.3)	2 (5.1)	0	33 (32.0)	4 (40.0)
Vascular disease	6 (5.9)	0	3 (7.7)	0	13 (12.6)	4 (40.0)
Mastectomy reason*						
Oncological	96 (68.1)	12 (66.7)	39 (75.0)	1 (33.3)	–	–
Mutation/prophylactic	4z (31.9)	6 (33.3)	13 (25.0)	2 (66.7)	–	–
Chemotherapy	66 (65.3)	5(41.7)	18 (46.1)	0	41 (39.8)	1(10.0)
Radiotherapy*	49 (34.8)	5 (27.8)	8 (15.4)	0	90 (74.4)	10 (76.9)
Endocrine therapy	47 (46.5)	4 (33.3)	16 (41.0)	1 (50.0)	55 (53.4)	4 (40.0)
Immunotherapy	10 (9.9)	0	5 (12.8)	0	12 (11.7)	0
Median surgical time [IQR], min	369 [280–429]	397 [291–519]	56 [39–70]	30 [13–47]	54 [39–65]	50 [38–75]
Mean hospital stay ± SD, days	5.4 ± 2.0	5.4 ± 3.1	1.2 ± 0.5	0.9 ± 0.2	1.0 ± 0.5	1.0 ± 0.3
Laterality						
Unilateral	61 (60.4)^#^	6 (50.0)^#^	26 (66.7)	0	85 (82.5)	7 (70.0)
Bilateral	40 (39.6)	6 (50.0)	13 (33.3)	2 (100.0)	18 (17.5)^##^	3 (30.0)^##^
Timing*						
Primary	53 (37.6)	10 (55.6)	43 (82.7)	3 (100.0)	–	–
Secondary	56 (39.7)	5 (27.8)	9 (17.3)	0	–	–
Tertiary	32 (22.7)	3 (16.7)	0	0	–	–

Values are expressed in N (%) unless otherwise specified

*BMI* body mass index

*Breasts as unit of analysis.

^#^52 conventional unilateral (48 COVID-negative, 4 COVID-positive) and 15 stacked unilateral (13 COVID-negative, 2 COVID-positive)

^##^4 after bilateral lumpectomy (3 COVID-negative, 1 COVID-positive), 17 after unilateral lumpectomy with immediate contralateral symmetrisation (15 COVID-negative, 2 COVID-positive)

### Surgical technique

**Fig. 1:**
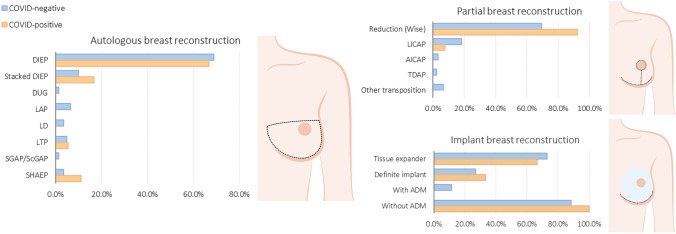
Surgical details. *DIEP* deep inferior epigastric perforator; *DUG* diagonal upper gracilis; *LAP* lumbar artery perforator; *LD* latissimus Dorsi; *LTP* lateral thigh perforator; *SGAP* superior gluteal artery perforator; *ScGAP* septocutaneous gluteal artery perforator; *SHAEP* stacked hemi-abdominal extended perforator. *ADM* acellular dermal matrix. *LICAP* lateral intercostal artery perforator; *AICAP* anterior intercostal artery perforator; *TDAP* thoracodorsal artery perforator

The surgical regimen represented in this cohort included a range of abdominal and non-abdominal based flaps in the ABR group, tissue expanders (72.7%) and definite implant (27.3%) in the IBR group, and Wise-pattern reduction mammoplasty as well as several local perforator-based transposition flaps in the PBR group. The ABR group consisted of 155 flaps in COVID-negative, and 22 in COVID-positive patients. The flap weight and ischemic time were similar in COVID-positive and –negative patients (691.2 ± 302.9 vs. 710.3 ± 264.8 g; 47 [35–70] vs. 43 [33–54] minutes). The median postoperative hospital stay was 5 days in the ABR group, versus 1 day for IBR and PBR. No clinically relevant differences in operative times between COVID-positive and COVID-negative patients were noted in all groups. The surgical techniques are summarized in Fig. 1.

### Complications

In the ABR group, intraoperative complications occurred in similar rates in COVID-positive versus COVID-negative (3 [16.7%] vs. 24 [17.0%]). The majority of intraoperative complications were vascular compromise requiring a revision (3 [16.7%] vs. 20 [14.2%]). When solely considering patients with preoperative COVID-infection (*n* = 6), the incidence of intraoperative complications remained similar. Complications per breast (major and minor pooled together) occurred evidently more often in the COVID-positive group (13 [72.2%] vs. 33 [23.4%], *p* < 0.001). Minor complications contributed most to this (11 [61.1%] vs. 23 [16.3%], *p* < 0.001). The incidence of major breast complications was slightly but not-significantly higher in COVID-positive patients. Half of the reinterventions were for vascular compromise (six times venous, two times arterial) and required reanastomosis. Other reasons for reintervention were hematoma, infection and partial flap necrosis. Three total and two partial flap losses occurred, all in the COVID-negative group (NS). Regarding minor breast complications, impaired wound healing and fat necrosis were observed more frequently in COVID-positive compared to COVID-negative patients (6 [33.3%] vs. 11 [7.8%], *p* = 0.004; 5 [27.8%] vs. 8 [5.7%], *p* = 0.009).

Donor site complications in the ABR cohort were observed more frequently in COVID-positive patients as well (6 [50.0%] vs. 25 [23.1%], *p* = 0.053). Specifically, the incidence of impaired wound healing was higher in the COVID-positive patients compared to COVID-negative (6 [50.0%] vs. 16 [14.8%], *p* = 0.008). Major donor site complications only occurred in COVID-positive patients and in none of the COVID-negative patients (2 [16.7%] vs. 0%, *p* = 0.009). 

In the PBR cohort, postoperative complications per breast were more often present in the COVID-positive group (10 [76.9%] vs. 31 [25.6%], *p* = 0.004). The vast majority were minor complications (9 [69.2%] vs. 24 [19.8%], p = 0.002). Major complications seemed more frequent in COVID-positive patients as well. However, this was not statistically significant (3 [23.1%] vs. 8 [6.6%], *p* = 0.185). Impaired wound healing was the most commonly observed complication and occurred evidently more often in COVID-positive patients (8 [61.5%] vs. 17 [14.0%], *p* = 0.007). The second most common complication was infection, which was also observed more often in COVID-positive patients (4 [30.8%] vs. 8 [6.6%], *p* = 0.008). Fat necrosis seems to be slightly more prevalent in COVID-positive patients as well, however this was not significant (2 [15.4%] vs. 2 [5.0%], *p* = 0.075).

Thromboembolic events occurred in 4 ABR patients, 0 IBR patients, and 1 PBR patient; all COVID-negative. Pulmonary complications occurred in one COVID-positive and two COVID-negative ABR patients, and one COVID-positive versus two COVID-negative PBR patients; both not statistically significant. There were no differences in pre- versus postoperative COVID-infection. In the IBR cohort, the incidence of COVID-19 was too low to perform statistical analysis. This was therefore omitted.

Complication rates are summarized in Table 2 and Fig. 2. A comprehensive overview including the absolute counts and percentages of all individual complications is added in Online Resource 1.

**Table 2:** Complications per breast

	COVID-negative	COVID-positive	Crude odds	P	Adj. odds^*^	*P*
Autologous breast reconstruction						
Breast complications	33 (23.4)	13 (72.2)	8.5 (2.8–25.6)	< 0.001	8.0 (2.7–24.4)	< 0.001
Major	12 (8.5)	3 (16.7)	2.2 (0.5–8.5)	0.275	2.3 (0.6–9.6)	0.248
Minor	23 (16.3)	11 (61.1)	8.1 (2.8–23.0)	< 0.001	7.4 (2.5–21.3)	< 0.001
Donor site complications^#^	25 (23.1)	6 (50.0)	3.3 (1.0–11.2)	0.053	3.3 (1.0–11.5)	0.059
Major^#^	0	2 (16.7)	N/A	0.009	N/A	N/A
Minor^#^	23 (21.3)	4 (33.3)	1.8 (0.5–6.7)	0.349	1.7 (0.4–6.2)	0.445
Reintervention	12 (8.5)	1 (5.6)	0.6 (0.1–5.2)	0.689	0.6 (0.1–5.1)	0.646
Reanastomosis	6 (4.3)	1 (5.6)	0.8 (0.2–11.7)	0.801	1.5 (0.2–13.2)	0.739
Partial breast reconstruction						
Breast complications	31 (25.6)	10 (76.9)	9.7 (2.5–37.5)	< 0.001	8.1 (1.9–34.0)	0.004
Major	8 (6.6)	3 (23.1)	4.3 (1.0–18.5)	0.055	3.0 (0.6–15.8)	0.185
Minor	24 (19.8)	9 (69.2)	9.1 (2.6–32.1)	< 0.001	8.1 (2.1–30.8)	0.002

Values presented as absolute count (proportion), odds ratio (95% confidence interval)

*Adjusted for age, BMI, radiotherapy and chemotherapy

#Donor sites as unit of analysis (108 COVID-, 12 COVID +)

**Fig. 2:**
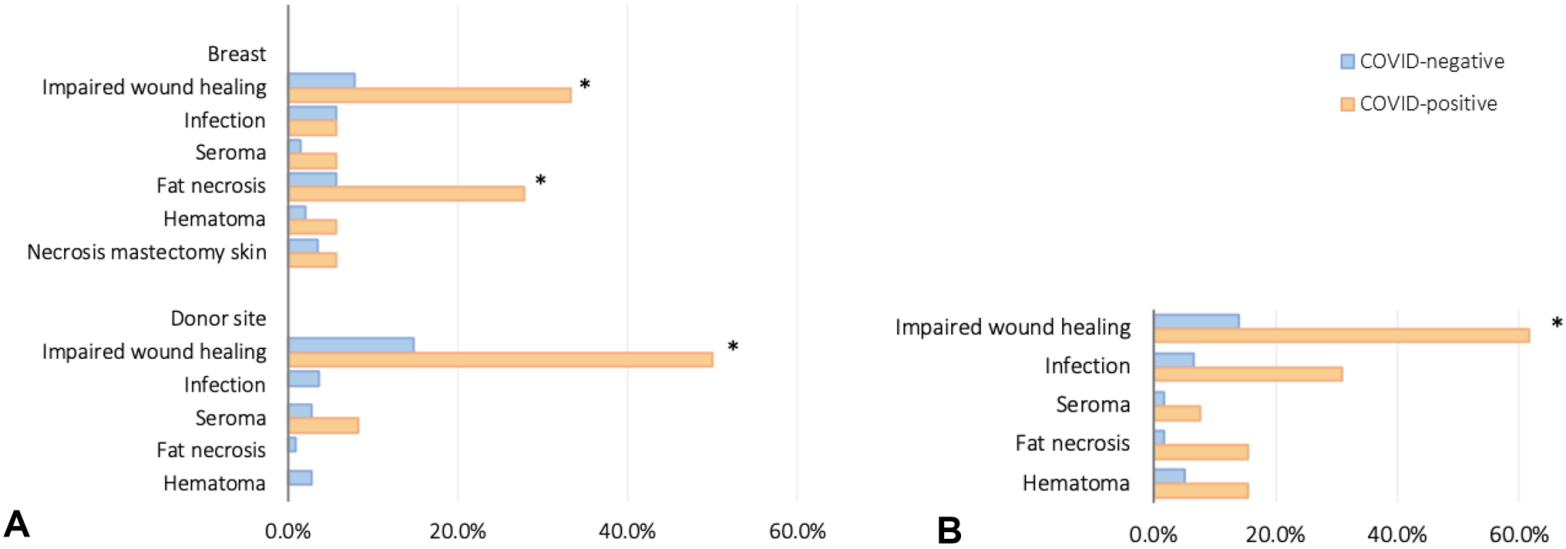
Postoperative complications in the ABR group (**A**), and PBR group (**B**)

## Discussion

The global COVID-19 pandemic has significantly impacted health care worldwide. Especially surgical care was drastically reformed during different phases of the pandemic, to spare clinical capacities and diminish disease transmissibility. The influence of COVID on surgical outcomes, especially related to postoperative complications in plastic surgery, are until now not entirely known. Therefore, this present study aimed to fill this knowledge gap.

This study focused on patients undergoing any type of reconstructive breast surgery. It must be noted that our IBR group was notably smaller than our ABR and PBR groups. This is due to the fact that the primary inclusion center of this study, Maastricht University Medical Center, is a renowned tertiary referral hospital for autologous breast reconstruction in the Netherlands.

The incidence of COVID-19 in the IBR group was remarkably low. Operative time, hospitalization and time to full recovery are markedly shorter in IBR patients compared to ABR. This possibly makes IBR patients less prone to attract COVID-19. While the same applies to PBR patients, this population is generally older and has more comorbidities; both increasing the vulnerability to a COVID-19 infection. Nevertheless, it is also well possible that the relatively low incidence of COVID-19 in IBR patients was due to the limited sample size.

The primary outcome of this study was the postoperative complication rate. In the ABR and PBR group, COVID-positive patients had a higher incidence of postoperative complications. Most complications were classified as minor, and did not require surgical intervention. A specifically notable finding was the significantly impaired wound healing at all surgical sites which we consistently found in the PBR group as well as the donor and recipient sites in the ABR group in COVID-positive compared to COVID-negative patients. Fat necrosis and surgical site infection also occurred more often in COVID-positive patients. Regarding donor site complications in the ABR group, a critical note is that this group consisted of a variation of different donor sites. Therefore, differences in postoperative recovery and complications may inherently be present. This may have influenced our findings, but could not be adjusted for due to the relatively low sample size in this study. Although our findings are statistically significant, it must be noted the 95% confidence intervals are wide. Therefore, we can conclude that our results suggest an association between COVID-19 and postoperative complications. However, the true effect size at population level may vary, as indicated by the wide 95% confidence intervals.

The clinical need for better understanding of COVID-19 and its implications on surgical care has led to an exponentially increasing scientific interest. Abundant evidence shows that perioperative COVID-infection elevates risks of cardiopulmonary, renal, septic and thromboembolic complications, as well as postoperative mortality across a wide range of surgical disciplines [12–18]. Therefore, anesthetic and surgical associations recommend postponing elective surgical procedures after COVID-infection [19–21]. While we did not observe increased mortality, or cardiopulmonary, renal or septic complications, it should be taken into account that the average breast reconstruction patient is relatively young and lacks significant comorbidities. Therefore, the patients included in our cohort are potentially less vulnerable than those in other published studies.

The pandemic evolved over time: different virus variants became endemic, and vaccinations became available. At this moment there is no evidence suggesting differences in surgical outcomes depending on the virus variant or pandemic phase [21, 22]. Contrarily, vaccination does contribute positively. This is partly due to a diminished disease risk, but secondly due to mitigation of the severity of the COVID-19 symptoms. Vaccinated COVID-positive patients have favorable surgical outcomes and lower complication rates compared to unvaccinated COVID-positive patients [23].

Although a vast amount of knowledge is available regarding surgical outcomes, preferable timing, and factors that affect postoperative recovery in COVID-positive patients; there is relatively little mention of surgical site complications. Nevertheless, a few studies and case series have shed light on this subject.

In cardiothoracic surgery several case series and a case–control study highlighted clinical cases of COVID-positive patients with anastomotic complications, wound disturbances and sternal dehiscence in patients after open cardiac surgery [24–26]. The described cases share all a component of unusualness: the complications developed sudden and without any accompanying signs, were more severe than usual, and often followed an uncommon course in time. The Cardiothoracic Interdisciplinary Research Network and COVIDSurg jointly confirmed that wound and sternal dehiscence occurs more often in patients with (especially postoperative) COVID-infection [27].

For oncological surgery a few case series described heightened risks for postoperative complications including impaired wound and anastomotic healing in COVID-positive patients as well [28, 29]. Similarly, unusual cases of wound dehiscence have been described in pressure ulcer surgery with local reconstruction.

Additionally, several reports of comparable complications have been published regarding flap reconstructions. Inouye et al. described two cases of free flap head and neck reconstruction, where both patients contracted COVID-19 postoperatively and presented with severe flap dehiscence and donor site skin graft failure [30]. In these cases, the unusual time span is again striking. The complications manifested at 16 and 20 days postoperatively, whereas in earlier stages the wound healing was unremarkable. Next, Chen et al. described a case of partial anterolateral thigh flap failure six weeks after surgery in a patient with active COVID-infection [31]. Talmor et al. published a case of complete necrosis of a pedicled nasoseptal flap for closure of a skull base defect four weeks postoperatively with concurrent COVID-19 infection, while the flap was still viable at endoscopic inspection two weeks postoperatively [32].And finally, Benmoussa et al. described a case centered around a chimeric double skin paddle-free fibula flap and a thoracodorsal artery perforator flap used for an intraoral defect, which both became necrotic synchronous with a COVID-19 infection one week postoperatively [33].

The findings of this present study in conjunction with the previously described literature suggests that patients with COVID-infection have an increased susceptibility to develop surgical site complications. They seem especially prone to impaired wound healing. This may be a result of inflammation and thrombosis in the surrounding microvasculature.

It is commonly acknowledged that COVID-19 affects microvasculature and causes aberrancies in haemostasis. A key role is reserved for angiotensin II (Ang II). Angiotensin converting enzyme 2 (ACE 2) is the functional receptor of the COVID-19 virus. ACE 2 is expressed strongly on endothelial cells, thereby providing an entry point for invasion by the virus. Binding to the ACE 2 receptor elicits Ang II production. Circulating levels of Ang II elevate as a result. Besides its vasoconstrictive properties, Ang II is also a pro-inflammatory mediator. It increases production of several other cytokines including antifibrinolytic mediators. This promotes (micro)vascular fibrin deposition and reduces demolition of thrombi [34–36].

Besides Ang II, also platelets presumably play a role in the disruption of haemostasis induced by COVID-19 [37]. In COVID patients, elevated levels of circulating platelets are observed; likely because the SARS-CoV-2 Spike protein directly stimulates platelets [38]. As platelets are an important element in primary haemostasis, this contributes further to a COVID-virus induced hypercoagulable state.

As wound healing and free flap survival rely heavily on adequate revascularization, they are dependent on surrounding microvasculature. It is therefore well imaginable that these coagulatory and inflammatory changes directly related to the COVID-19 virus have potential to negatively influence surgical site outcomes. Interestingly, while we indeed observed an increased susceptibility to wound healing disturbances, we did not see decreased flap survival or increased vascular compromise. Additionally, the incidence of thromboembolic events was not increased. Possibly, the routine administration of postoperative low-molecular weight heparin prevented this from occurring in our cohort. Alternatively, it is possible that merely patients with a preoperative COVID-19 infection are prone to these complications and our proportion of preoperative COVID-positive patients may have been too small to observe this.

This study is the first to shed light on postoperative outcomes and complications associated with COVID-19 after reconstructive breast surgery. Unfortunately, the IBR group was relatively small and had a lower incidence of COVID than the ABR and PBR groups. Therefore, no statistical testing could be conducted which limits our results of this group. Future research with larger sample sizes would be valuable to assess whether our findings in ABR and PBR patients can be extrapolated to IBR patients as well. A larger sample size would also provide additional power to confirm our results. Furthermore, with larger sample sizes pre- and postoperative COVID-infection can be more reliably compared to identify if either increases the risk of postoperative complications more. Another arguable limitation is that we did not take into account vaccination status in this study. Although vaccinations protect against perioperative mortality and morbidity, it remains unknown whether this also accounts for surgical site complications. Additional research into the hypothesized underlying pathophysiological mechanisms is also recommended to better understand how COVID affects postoperative recovery and develop risk-reducing strategies. Histopathological examination of tissue samples from patients with for example disturbed wound healing could reveal valuable insight regarding the pathophysiology.

## Conclusion

This study indicates that COVID-19 is associated with elevated risks for postoperative complications after reconstructive breast surgery. In our cohort this was clearly indicated by a significantly higher incidence of minor and major complications, especially impaired wound healing in patients after autologous breast reconstruction or partial breast reconstruction.

Clinically, we recommend to postpone elective surgery according to current guidelines. Medical personnel should carefully aim to reduce viral transmission to a large extent. And importantly, we recommend informing the patients and their relatives about the postoperative risks associated with COVID-19. They should be advised to take precautionary measures to minimize the risk of contracting COVID-19 in the weeks subsequent to breast cancer surgery.

## Supplementary Information

Below is the link to the electronic supplementary material.

Supplementary file1 (DOCX 17 KB)

## Data Availability

The datasets generated during and/or analyzed during the current study are available from the corresponding author on reasonable request.
