# Supplementary material for: COVID-associated complications after reconstructive breast surgery: a retrospective cohort study
Source: Breast Cancer Res Treat. 2023 Jul 29;202(2):257–65. doi: 10.1007/s10549-023-07064-1 (PMC10505595; doi:10.1007/s10549-023-07064-1)
Supplement: Supplementary file 1 — Supplementary file1 (DOCX 17 KB) [file 10549_2023_7064_MOESM1_ESM.docx]

**Online Resource 1: complication rates**

**Table 1.** Complications specified per breast and donor site in patients with autologous breast reconstruction

|  | **COVID-negative** | | **COVID-positive** | | **Crude odds**  **(95% CI)** | | **P** | **Adj. odds**  **(95% CI)** | | **P** |
| --- | --- | --- | --- | --- | --- | --- | --- | --- | --- | --- |
| **BREAST** |  |  |  |  |  |  |  |  |  |  |
| Total flap loss | 3 | (2.1) | 0 |  |  |  |  |  |  |  |
| Partial flap loss | 2 | (1.4) | 0 |  |  |  |  |  |  |  |
| Venous insufficiency | 5 | (3.5) | 1 | (5.6) | 1.6 | (0.2-14.5) | 0.676 | 1.7 | (0.2-16.0) | 0.653 |
| Arterial insufficiency | 1 | (0.7) | 1 | (5.6) | 8.2 | (0.5-137.8) | 0.142 | 7.8 | (0.4-137.2) | 0.160 |
| Impaired wound healing | 11 | (7.8) | 6 | (33.3) | 5.9 | (1.9-18.8) | 0.003 | 5.9 | (1.7-19.6) | 0.004 |
| Infection | 8 | (5.7) | 1 | (5.6) | 1.0 | (0.1-8.3) | 0.984 | 0.6 | (0.1-5.9) | 0.648 |
| Hematoma | 3 | (2.1) | 1 | (5.6) | 2.7 | (0.3-27.5) | 0.400 | 3.0 | (0.3-32.2) | 0.375 |
| Fat necrosis | 8 | (5.7) | 5 | (27.8) | 6.4 | (1.8-22.4) | 0.004 | 5.5 | (1.5-20.0) | 0.009 |
| Seroma | 2 | (1.4) | 1 | (5.6) | 4.1 | (0.4-47.5) | 0.260 | 4.4 | (0.4-54.0) | 0.245 |
| Necrosis mastectomy skin | 5 | (3.5) | 1 | (5.6) | 1.6 | (0.2-14.5) | 0.676 | 1.5 | (0.2-13.7) | 0.745 |
| **DONOR SITE** |  |  |  |  |  |  |  |  |  |  |
| Infection | 4 | (3.7) | 0 |  |  |  |  |  |  |  |
| Hematoma | 3 | (2.8) | 0 |  |  |  |  |  |  |  |
| Seroma | 3 | (2.8) | 1 | (8.3) | 3.2 | (0.3-33.3) | 0.334 | 2.9 | (0.3-32.1) | 0.375 |
| Fat necrosis | 1 | (0.9) | 0 |  |  |  |  |  |  |  |
| Impaired wound healing | 16 | (14.8) | 6 | (50.0) | 5.8 | (1.6-20.1) | 0.006 | 6.0 | (1.6-22.6) | 0.008 |

Data are presented as N (%)

**Table 2.** Complications per breast in patients with implant-based breast reconstruction.

|  | **COVID-negative** | | **COVID-positive** | | **Crude odds (95% CI)** | | **P** | **Adj. odds**  **(95% CI)** | | **P** |
| --- | --- | --- | --- | --- | --- | --- | --- | --- | --- | --- |
| Infection | 5 | (9.6) | 0 |  |  |  |  |  |  |  |
| Seroma | 11 | (21.2) | 2 | (66.7) | 7.5 | (0.6-90.0) | 0.114 | 3.6 | (0.2-57.8) | 0.371 |
| Necrosis mastectomy skin | 3 | (5.8) | 0 |  |  |  |  |  |  |  |
| Implant leakage | 1 | (1.9) | 2 | (66.7) | 102.0 | (4.5-2289) | 0.004 | * |  | 0.998 |
| Re-intervention, due to  Implant leakage  Necrosis  Infection | 8  1  3  4 | (15.4)  (1.9)  (5.8)  (7.7) | 2  2  0  0 | (66.7)  (66.7) | 11.0 | (0.9-136.2) | 0.062 | 4.2 | (0.2-76.9) | 0.339 |

Data are presented as N (%)

*due to low incidence, no adjusted odds ratio and 95% confidence interval could be computed.

**Table 3.** Complications per breast in patients with partial breast reconstruction.

|  | **COVID-negative** | | **COVID-positive** | | **Crude odds (95% CI)** | | **P** | **Adj. odds (95% CI)** | | **P** |
| --- | --- | --- | --- | --- | --- | --- | --- | --- | --- | --- |
| Impaired wound healing | 17 | (14.0) | 8 | (61.5) | 9.8 | (2.9-33.5) | <0.001 | 6.1 | (1.6-22.6) | 0.007 |
| Infection | 8 | (6.6) | 4 | (30.8) | 6.3 | (1.6-24.9) | 0.009 | 8.8 | (1.8-43.6) | 0.008 |
| Hematoma | 6 | (5.0) | 2 | (15.4) | 3.5 | (0.6-19.4) | 0.154 | 4.5 | (0.7-30.8) | 0.121 |
| Fat necrosis | 2 | (1.7) | 2 | (15.4) | 10.8 | (1.4-84.5) | 0.023 | 9.0 | (0.8-101.4) | 0.075 |
| Seroma | 2 | (1.7) | 1 | (7.7) | 5.0 | (0.4-58.8) | 0.204 | * |  | 0.996 |
| Re-intervention | 9 | (7.4) | 3 | (23.1) | 3.7 | (0.9-16.0) | 0.077 | 2.3 | (0.4-11.8) | 0.323 |

Data are presented as N (%)

*due to low incidence, no adjusted odds ratio and 95% confidence interval could be computed.
